# Waiting for the Pediatric Acute Lung Injury Consensus Conference 3

**DOI:** 10.62675/2965-2774.20240114-en

**Published:** 2024-06-05

**Affiliations:** 1 Hospital Israelita Albert Eintein São Paulo SP Brazil Hospital Israelita Albert Eintein - São Paulo (SP), Brazil.; 2 Hospital da Luz Vila Mariana São Paulo SP Brazil Hospital da Luz Vila Mariana - São Paulo (SP), Brazil.; 3 FIOCRUZ Instituto Nacional da Saúde da Mulher, da Criança e do Adolescente Fernandes Figueira Rio de Janeiro RJ Brazil Instituto Nacional da Saúde da Mulher, da Criança e do Adolescente Fernandes Figueira - FIOCRUZ - Rio de Janeiro (RJ), Brazil.; 4 Instituto D'Or de Pesquisa e Ensino Rio de Janeiro RJ Brazil Instituto D'Or de Pesquisa e Ensino - Rio de Janeiro (RJ), Brazil.

Since the publication of a case series by Ashbaugh et al. in 1967 involving 11 adult patients and only one child, pediatricians have been trying to define acute respiratory distress syndrome (ARDS) in pediatric patients.^(1)^ In 1988, Murray et al. created a score for the classification of ARDS using four variables—chest radiography, partial pressure of oxygen to fraction of inspired oxygen (PaO_2_/FiO_2_), end-expiratory airway pressure (PEEP) and lung compliance—but only for adult patients.^(2)^ Six years later, in 1994, the American-European Consensus Conference (AECC) published the first definition of ARDS, using PaO_2_ /FiO_2_, regardless of PEEP, again excluding pediatric patients.^(3)^ In 2012, a new definition was published resulting from a consensus conducted in the city of Berlin, Germany, using PaO_2_/FiO_2_ and the PEEP level, which also did not include children or adolescents.^(4)^ Nevertheless, pediatricians have begun to adopt the Berlin criteria to define ARDS in the absence of a proper definition. Both definitions of ARDS, that of the AECC and that of Berlin, focus on lung injury in adults and have limitations when applied to children. For example, an important deficiency is the need for invasive measurement of arterial oxygen, which can become a challenge in infants and children who are agitated or uncooperative. A second limitation is the use of PaO_2_ /FiO_2_. In addition to the need for the measurement of PaO_2_, this relationship is influenced by ventilator pressure. Consequently, differences in pediatric clinical practice may influence the diagnosis, particularly because there is greater variability in ventilatory parameters in pediatric intensive care units (ICUs) than in adult ICUs.

In 2015, the idea of a pediatric definition was promoted by the Pediatric Acute Lung Injury and Sepsis Investigators (PALISI), which was later supported by several other research groups around the world. Thus began the Pediatric Acute Lung Injury Consensus Conference (PALICC), in which PaO_2_ /FiO_2_ was replaced by the oxygenation index (FiO_2_ x mean airway pressure/partial arterial oxygen pressure) and oxygen saturation index (FiO_2_ x mean airway pressure/oxygen saturation), including the management of the patient with pressure on mechanical ventilators, in addition to including a measurement not dependent on arterial blood gas analysis.^(5)^ In 2023, an improved definition was published, PALICC-2, which started to include noninvasive ventilation modes, among other changes.^(6)^

Why are such definitions so important? Valid and reliable definitions are essential for successfully conducting epidemiological studies and facilitating the inclusion of a consistent patient phenotype in clinical trials. Intensivists also need such definitions to implement interventions, observe risk factors and predict outcomes, as well as discuss prognosis with families and plan resource allocation.

The study by Capela et al.,^(7)^ published in Critical Care Science, compared two methods for defining and classifying pediatric ARDS severity: the Berlin classification, which uses PaO_2_/FiO_2_, and the PALICC classification, which uses the oxygenation index. The authors found significant agreement between the classifications of pediatric ARDS severity (Berlin and PALICC), accompanied by a very strong numerical correlation between PaO_2_/FiO_2_ and the oxygenation index, as well as the presence of a significant effect of neuromuscular blockers on these classifications. Interestingly, there was higher agreement between the classifications in patients not using neuromuscular blockers, suggesting its influence on the classifications of ARDS severity. In addition, the PaO_2_/FiO_2_ values could change significantly depending on how the mechanical ventilator was adjusted in patients with ARDS, although the oxygenation index remained constant.

The treatment of ARDS is controversial, and there is a lack of evidence, with few randomized controlled trials in the pediatric population. Thus, a robust and validated definition for the pediatric population is extremely relevant for conducting intervention studies and identifying the best clinical practices for pediatric ARDS patients. Using the PALICC criteria to classify pediatric patients with moderate/severe ARDS, the international study PROSPect (prospect-network.org), which compares conventional mechanical ventilation to high-frequency oscillatory ventilation, in addition to comparing the supine and prone positions, is being conducted in 56 pediatric ICUs.^(5)^ The primary outcome studied will be free days of mechanical ventilation. In the future, we will likely have a robust study with evidence on how to better manage our patients diagnosed with ARDS.

In addition to the study that is being conducted, epidemiological studies, such as PARDIE, are essential to support our clinical practice and improve the definitions of ARDS.^(8)^ In this regard, the article by Capela et al.^(7)^ helps us to evaluate the practical implementation of the definitions of ARDS in real clinical scenarios of pediatric patients in a Brazilian unit. Despite this being a single-center, observational study that is based on a convenience sample and has other methodological limitations, the authors shed light on the complexity of the evaluation of ARDS in pediatric patients, taking into account the clinical conditions and the drugs in use when interpreting the criteria for ARDS severity. Capela et al.^(7)^ raise relevant questions and highlight the need for further refinement of the definition of ARDS, taking into account the use of neuromuscular blocking agents, a practice that is still common in pediatric units.

While we await the PALICC-3, new methodologically robust and collaborative studies are needed for a better understanding of ARDS and to reduce morbidity and mortality associated with this challenging condition in pediatric patients.
