# Supplementary material for: Waiting for the Pediatric Acute Lung Injury Consensus Conference 3
Source: Crit Care Sci. 2024 Jun 5;36:e20240114en. doi: 10.62675/2965-2774.20240114-en (PMC11152441; doi:10.62675/2965-2774.20240114-en)
Supplement: Translation

# Translation: À espera da *Pediatric Acute Lung Injury Consensus Conference 3*

**DOI:** 10.62675/2965-2774.20240114-pt

**Affiliations:** 1 São Paulo SP Brasil Hospital Israelita Albert Eintein - São Paulo (SP), Brasil.; 2 São Paulo SP Brasil Hospital da Luz Vila Mariana - São Paulo (SP), Brasil.; 3 Rio de Janeiro RJ Brasil Instituto Nacional da Saúde da Mulher, da Criança e do Adolescente Fernandes Figueira - FIOCRUZ - Rio de Janeiro (RJ), Brasil.; 4 Rio de Janeiro RJ Brasil Instituto D'Or de Pesquisa e Ensino - Rio de Janeiro (RJ), Brasil.

Desde a publicação de uma série de casos por Ashbaugh et al. em 1967, com 11 pacientes adultos e apenas uma criança, os pediatras vêm tentando definir o que seria a síndrome do desconforto respiratório agudo (SDRA) na pediatria.^(1)^ Somente em 1988, Murray et al. criaram um escore para classificação da SDRA, utilizando quatro variáveis: radiografia de tórax, razão entre pressão parcial de oxigênio e fração inspirada de oxigênio (PaO_2_/FiO_2_), pressão expiratória final de vias aéreas (PEEP) e complacência pulmonar, porém apenas para pacientes adultos.^(2)^ Seis anos depois, em 1994, a *American-European Consensus Conference* (AECC) publicou a primeira definição de SDRA, utilizando PaO_2_/FiO_2_, independentemente da PEEP, deixando novamente de fora a pediatria.^(3)^ Em 2012, foi publicada uma nova definição resultante de um consenso realizado na cidade de Berlim, na Alemanha, utilizando PaO_2_/FiO_2_ e também o nível da PEEP, que também não incluiu crianças ou adolescentes.^(4)^ Independentemente disso, os pediatras começaram a adotar os critérios de Berlin para definir SDRA, na falta de uma definição própria. Ambas as definições de SDRA, a da AECC e a de Berlim, concentram-se na lesão pulmonar em adultos e apresentam limitações quando aplicadas a crianças. Por exemplo, uma deficiência importante é a necessidade de medição invasiva de oxigênio arterial, o que pode se tornar um desafio em lactentes e crianças com agitação ou pouco cooperativas. Uma segunda limitação é a utilização da PaO_2_/FiO_2_. Além de exigir a medição da PaO_2_, essa relação é influenciada pelas pressões do ventilador. Consequentemente, diferenças na prática clínica pediátrica podem influenciar o diagnóstico, particularmente por haver maior variabilidade nos parâmetros ventilatórios, com relação às unidades de terapia intensiva (UTIs) de adultos.

Somente em 2015, a ideia de uma definição pediátrica foi impulsionada pelo *Pediatric Acute Lung Injury and Sepsis Investigators* (PALISI), sendo posteriormente apoiada por diversos outros grupos de pesquisa pelo mundo. Assim surgiu a *Pediatric Acute Lung Injury Consensus Conference* (PALICC), no qual PaO_2_/FiO_2_ foi substituído pelo índice de oxigenação (FiO_2_ x pressão média de vias aéreas/pressão arterial parcial de oxigênio) e pelo índice de saturação de oxigênio (FiO_2_ x pressão média de vias aéreas/saturação de oxigênio), contemplando o manejo do paciente com as pressões no ventilador mecânico, além de incluir uma medida não dependente da gasometria arterial.^(5)^ Em 2023, um aprimoramento da definição foi publicado, a PALICC-2, que passou a incluir modos de ventilação não invasiva, dentre outras alterações.^(6)^

E por que tais definições são tão importantes? Definições válidas e confiáveis são essenciais para conduzir estudos epidemiológicos com sucesso e facilitar a inclusão de um fenótipo de paciente consistente em ensaios clínicos. Intensivistas também precisam de tais definições para implementar intervenções, observar fatores de risco e predizer desfechos, bem como discutir o prognóstico com as famílias e planejar a alocação de recursos.

O estudo de Capela et al.,^(7)^ publicado na *Critical Care Science*, compara dois métodos para definição e classificação de gravidade na SDRA pediátrica: a classificação de Berlim, que utiliza PaO_2_/FiO_2_, e a classificação PALICC, que utiliza o índice de oxigenação. Os autores encontraram concordância significativa entre as classificações de gravidade da SDRA pediátrica (Berlim e PALICC), acompanhada de correlação numérica muito forte entre PaO_2_/FiO_2_ e o índice de oxigenação, bem como a presença de efeito significativo do bloqueador neuromuscular sobre essas classificações. Interessante observar que houve maior concordância entre as classificações nos pacientes sem uso do bloqueador neuromuscular, sugerindo influência dele sobre as classificações de gravidade da SDRA. Além disso, a depender de como era feito o ajuste do ventilador mecânico no paciente com SDRA, os valores da PaO_2_/FiO_2_ podiam alterar bastante, embora o índice de oxigenação permanecesse constante.

O tratamento da SDRA é envolto em controvérsias e falta de evidências, com poucos estudos randomizados controlados em pediatria. Dessa forma, uma definição robusta e validada para a população pediátrica é de extrema relevância para a condução de estudos de intervenção e identificação de melhores práticas clínicas na SDRA pediátrica. Utilizando os critérios da PALICC para classificar pacientes pediátricos com SDRA moderada/grave, o estudo internacional PROSPect (prospect-network.org), que compara ventilação mecânica convencional *versus* ventilação oscilatória de alta frequência, além da posição supina *versus* posição prona, está sendo conduzido em 56 UTIs pediátricas.^(5)^ O desfecho primário estudado será dias livres de ventilação mecânica. Em alguns anos, possivelmente, teremos um estudo robusto com evidências de como manejar melhor nossos pacientes com diagnóstico de SDRA.

Além do estudo que está sendo realizado, estudos epidemiológicos, como o PARDIE, são fundamentais para alicerçar nossa prática clínica e aprimorar as definições de SDRA.^(8)^ Nesse sentido, o artigo de Capela et al.^(7)^ nos auxilia a avaliar a implementação prática das definições de SDRA em cenários clínicos reais de pacientes pediátricos em uma unidade brasileira. Apesar de ser unicêntrico, observacional, baseado em uma amostra de conveniência e apresentar outras limitações metodológicas, os autores lançam luz sobre a complexidade da avaliação da SDRA em pacientes pediátricos, levando em consideração as condições clínicas e os medicamentos em uso ao interpretar os critérios de gravidade da doença. Capela et al.^(7)^ trazem questionamentos relevantes e destacam a necessidade de um novo aprimoramento da definição de SDRA, levando em consideração a utilização de bloqueadores neuromusculares, prática ainda comum em unidades pediátricas.

Enquanto estamos à espera da PALICC-3, novos estudos metodologicamente robustos e colaborativos são necessários para melhor compreensão da SDRA e redução da morbimortalidade associada a essa condição desafiadora nos pacientes pediátricos.
